# Anxiolytic and Antioxidant Effect of Phytoecdysteroids and Polyphenols from *Chenopodium quinoa* on an In Vivo Restraint Stress Model

**DOI:** 10.3390/molecules27249003

**Published:** 2022-12-17

**Authors:** Yuliya S. Sidorova, Vladimir A. Shipelin, Nikita A. Petrov, Sergey N. Zorin, Vladimir K. Mazo

**Affiliations:** Federal Research Centre of Nutrition and Biotechnology, 109240 Moscow, Russia

**Keywords:** quinoa, stress, polyphenols, Wistar rats, ICR mice, immobilization, memory, anxiety, catecholamines, acute toxicity

## Abstract

The variety of stressful conditions in daily human activity requires nutritional support with safe, specialized food products containing functional food ingredients (FFIs) enriched with biologically active plant substances with proven adaptogenic properties. In this in vivo study, by evaluating a set of physiological parameters and biochemical markers, we investigated the effectiveness of the developed FFIs from *Chenopodium quinoa* grains in stress conditions induced by daily episodes of immobilization for 36 days. The results of the evaluation of the anxiety-like functions, locomotor, and search activity of rats in the “open field” and “elevated plus maze” tests demonstrated the ability of FFIs to reduce stressful behavior induced by immobilization. The improvement in the long-term memory of animals treated with FFIs was noted in the passive avoidance test. Together with the hypolipidemic effect and compensation of transaminase levels, FFIs normalized the excretion of catecholamines in the urine and reduced the levels of malondialdehyde to values of the control group. According to the results of the assessment of FFI acute oral toxicity, the LD_50_ value exceeded 5000 mg/kg of body weight, which categorizes the FFIs under hazard class 5—substances with low hazard. The conducted experiment demonstrated the effectiveness of nutritional support with FFIs on the selected stress model. The positive safety profile of FFIs makes them reasonable to study on other stress models and to conduct clinical testing as part of specialized food products in various categories of people exposed to chronic stress.

## 1. Introduction

Human activity related to increased psychoemotional and physical load, harmful working conditions, or adverse climatic conditions requires constant improvement of personalized nutrition programs. The prospective approach is to use specialized food products (SFPs), containing functional food ingredients (FFIs) with proven adaptogenic action against the stress of various genesis [1]. Minor biologically active plant substances (BASs), adaptogens used in nutrition, provide an increase of human organism resistance to adverse influence and stress [2]. A prospective source of a wide range of phytoecdysteroids and polyphenols of adaptogenic action is quinoa (*Chenopodium quinoa* Willd.) grains [3,4]. The polyphenolic composition of black, white, red, and yellow quinoa is represented by more than 90 compounds of various natures: flavonoids, anthocyanins, dihydroflavones, dihydroflavonols, isoflavones, flavonols, and chalcones [5]. Among the main polyphenolic compounds in the composition of quinoa grains are vanillic, t-ferulic, protocatechuic, 4-hydroxybenzoic, o-cumaric, 3,4-dimetoxicinnamic, gallic acids, catechin and epicatechin, rutin, quercetin-3-O-rutinoside and quercetin-3-O-glucoside, kaempferol-3-O-glucoside and kaempferol-3-O-rutinoside, daidzein, and genistein [6,7]. Polyphenols in the composition of quinoa grains are presented in both free and conjugated forms [8].

The great diversity of polyphenolic compounds in the composition of quinoa grains determines the wide range of its biological activity. The nutritional value and the methionine and lysine content of quinoa protein are similar to milk casein, and the content of dietary fibers, vitamins (B1, B2, B6, C, E), and minerals (calcium, phosphorus, iron, and zinc) is higher than in cereals [9]. Quinoa has pronounced antioxidant activity, as shown in in vitro studies [10,11] or in in vivo experiments, where quinoa consumption showed antihyperlipidemic action against the background of a high-fat diet, leading to increased superoxide dismutase and glutathione peroxidase activities and to decreased levels of malondialdehyde [12] and inflammatory cytokines [13,14]. Sinapinic acid in the composition of quinoa grains can potentiate GABA currents in individual cortical neurons and thus exert pronounced anxiolytic effects [8,15].

The hormone 20-hydroxyecdysone (20E) and its structural analogs present in quinoa grain show a wide range of adaptogenic effects. The consumption of phytoecdysteroids provides anabolic effects and an increase in physical capability in the absence of androgenic and estrogenic effects [16,17,18], anxiolytic and antidepressant effects during immobilization stress [19], the normalization of oxidative phosphorylation process in mitochondria during immobilization stress [20], and an improvement in cognitive functions and spatial learning [21].

Of special interest is the study of synergistic effects of phytoecdysteroids and polyphenols in the composition of quinoa grains. Modern technological approaches, including the targeted extraction and concentration of complexes with a required ratio of phytoecdysteroids and polyphenolic compounds, together with the technologies of the ultrafiltration of low-molecular fractions, allow achieving high concentrations of BASs in the composition of FFIs. The absence of gliadins in quinoa makes it prospective for inclusion into the composition of SFPs for people with celiac disease [9]. The development of the FFIs of adaptogenic action requires an evaluation of their correspondence to the criteria of safety and effectiveness in preclinical in vivo studies and, further, in clinical tests.

The study aimed to accomplish the complex physiological and biochemical evaluation of the nutritional effectiveness of phytoecdysteroids and other minor BASs from quinoa grains in the formation of a nonspecific resistance of rat organism to emotional stress induced by forced immobilization.

## 2. Results

### 2.1. Acute Oral Toxicity of FFIs from Chenopodium quinoa

A single oral administration of developed FFIs from *Chenopodium quinoa* in a dose of 5000 mg/kg of body weight didn’t cause animal death. During the first four hours after FFI administration (Supplementary Table S1) and the following 13 days, all mice were motile; had normal appearance, feces, and food consumption; and had similar body weight gain. The autopsy on the 14th day of the experiment didn`t show any pathologic changes in internal organs. No adverse changes were found in body weight gain, relative liver weight, liver lipid profile, corticosterone, and prostaglandin E2 plasma levels of experimental animals in comparison with control ones (Supplementary Table S2).

Thus, it was not possible to determine the LD_50_ of FFIs for mice from the results of this experiment. We suppose that the LD_50_ value is higher than 5000 mg/kg of body weight, which allows classifying FFIs from *Chenopodium quinoa* as low toxicity substances (hazard class 5) [22].

### 2.2. 36-Day Study of the Quinoa FFI Adaptogenic Properties in a Model of Immobilization-Induced Emotional Stress

#### 2.2.1. Integral Indicators

The results of preliminary animals’ randomization according to their behavioral phenotype in the elevated plus maze (EPM) test and other integral parameters on the first experimental day are shown in Table 1.

**Figure 1:**
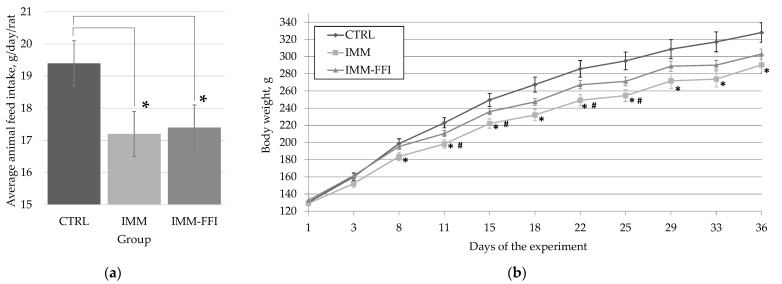
Integral indicators of rats during experiment No. 1: (**a**) average animal feed intake, g/day/rat; (**b**) average rats weight gain, g. *—differences are significant compared with the CTRL group, *p* < 0.05. **#**—differences are significant compared with the IMM-FFI group, *p* < 0.05.

The general condition of animals according to their appearance, behavior, feces, fur quality, food, and water intake monitored daily throughout the experiment was satisfying. Average food intake by animals during the experiment is presented in Figure 1a. The average food intake of IMM and IMM-FFI groups’ animals stressed with daily immobilization was significantly lower than that of CTRL group animals (*p* < 0.05, Mann–Whitney test). The calculated intake of 20E in the composition of FFIs by animals of the IMM-FFI group was 1.72 ± 0.02 mg/kg b.w./day; the intake of flavonoids was 9.37 ± 0.14 mg/kg b.w./day. Starting from the eighth day of the experiment, the average body weight of IMM group animals was significantly lower than that of CTRL group animals. Starting from the 11th day until the 25th day of the experiment, the body weight of IMM group animals was also significantly lower than that of IMM-FFI group animals receiving FFIs. At the same time, the body weight of the IMM-FFI group animals did not differ significantly from that of the CTRL group animals. However, starting from the eighth day of the experiment, the body weight gain of all animals exposed to forced immobilization was significantly lower than that of CTRL group animals. The consumption of FFIs neutralized to some degree the adverse effects of immobilization on the body weight of stressed animals.

#### 2.2.2. Memory Function and Behavioral Responses

**Figure 2:**
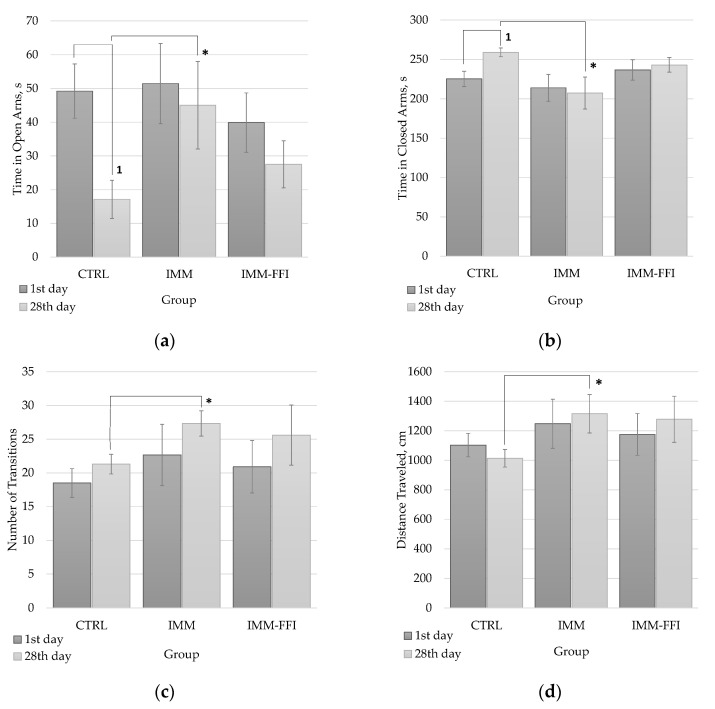
EPM testing results: (**a**) time in open arms, s; (**b**) time in closed arms, s; (**c**) a number of transitions between maze arms; (**d**) distance traveled in the maze, cm. First test before feeding by experimental rations. Second test on the 28th day of the experiment. 1—differences are significant (*p* < 0.05) compared with 1st test. *—differences are significant compared with the CTRL group, *p* < 0.05.

Figure 2 shows the results of testing in EPM at the beginning of the experiment and after 28 days. As can be seen from the data presented in Figure 2a,b at the first test, there were no significant differences in the time intervals spent by animals in the open and closed arms of the maze, characterizing the anxiety-like functions of animals. Significant differences in motor and research activity, estimated by the indicators of movement between the arms of the maze (Figure 2c) and the total distance traveled (Figure 2d), were absent between the compared groups at the beginning of the experiment. During repeated testing on the 28th day of the experiment, the behavior of the animals changed. Animals of the CTRL group spent significantly less time in the open arms of the maze (Figure 2a) and significantly more in the closed arms (Figure 2b) than in the first test, which, in general, is consistent with age-related changes in animal behavior. However, no such changes in behavior were detected for groups exposed to immobilization. The rats of the stressed IMM group spent significantly more time in the open arms and significantly less in the closed arms of the maze than the CTRL control group did. A similar result was revealed in the indicators of locomotor activity (Figure 2c,d): the total distance traveled and the number of transitions between the arms of the maze were significantly greater in animals of the IMM group than control animals of the CTRL group. For stressed animals of the IMM-FFI group receiving FFIs, there were no significant differences between the CTRL and IMM comparison groups.

**Figure 3:**
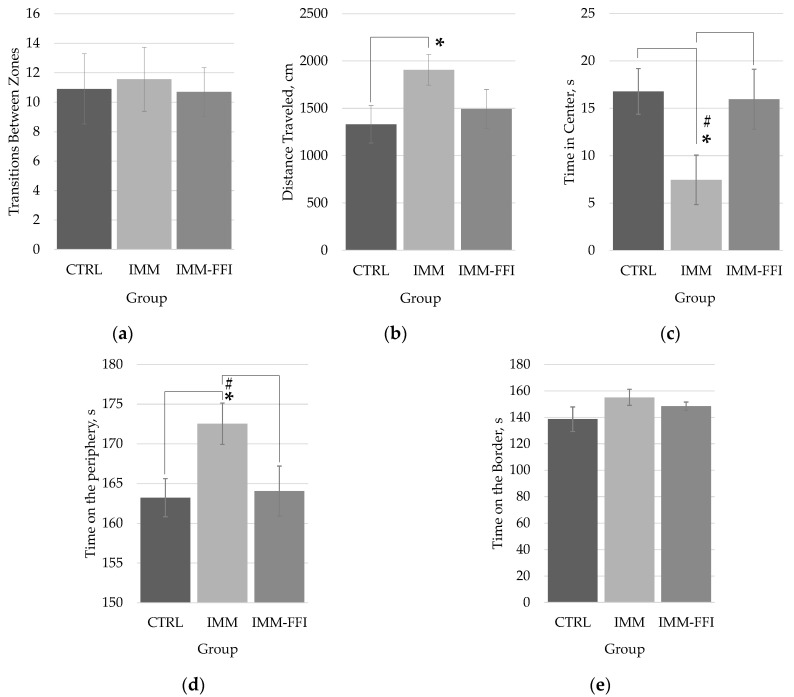
OF testing results on 22nd day of the experiment: (**a**) a number of transitions between zones; (**b**) distance traveled, cm; (**c**) time spent in the central zone, s; (**d**) time spent on the periphery, s; (**e**) time spent on the border, s. *—differences are significant compared with the CTRL group, *p* < 0.05. **#**—differences are significant compared with the IMM-FFI group, *p* < 0.05.

Figure 3 shows the results of testing in the open field (OF) test on the 22nd day of the experiment. The results showed the absence of significant differences in the behavior of animals of the experimental group IMM-FFI treated with FFIs, compared with intact animals of the control group CTRL. According to the indicators of anxiety-like functions (Figure 3b–e), it was found that the animals of the IMM group spent significantly (*p* < 0.05, Mann–Whitney test) less time in the center of the maze, staying mainly in the periphery zone compared with the rats of the CTRL and IMM-FFI groups, thus demonstrating increased anxiety-like functions. At the same time, the animals of the IMM group moved significantly more through the maze (Figure 3b) than did the rats of the CTRL group, which is also consistent with the increased motor activity detected in the EPM test (Figure 2c,d). The combined use of two physiological tests, EPM and OF, showed that the inclusion of FFIs in the diet leveled the effect of immobilization stress on the behavioral indicators of animals.

Assessment of short-term and long-term memory, as well as the cognitive functions of rats, were evaluated in the CPAR test (Table 2). During the first testing, the development of conditioned reflex of passive avoidance, animals of all groups entered the dark compartment of the camera (100% reflex production). On the 2nd day of testing, there were no significant differences in short-term memory between the groups. On the 14th day of long-term memory testing, an increase in the latency period of entry of animals of the IMM-FFI group into the dark compartment of the camera was found, along with a decrease in the number of animals that failed the test, compared with the CTRL control group. The obtained result indicates an improvement in the long-term memory of animals receiving FFIs against the background of immobilization stress.

#### 2.2.3. Biochemical Indices

Table 3 shows the results of determining the content of protein, lipid, purine, and mineral metabolism in the blood plasma of rats, indicators of the functional state of the liver and of blood glucose levels. The result of the stressful effect of daily forced immobilization was a significant increase in the level of corticosterone in rats of the IMM and IMM-FFI groups compared with animals of the CTRL control group. The introduction of FFIs obtained from quinoa grains into the diet did not have a compensatory effect on the level of corticosterone, the main biomarker of stress in the blood of animals. Chronic immobilization caused a significant increase in total bilirubin levels in the IMM group compared with the CTRL group. At the same time, in animals of the IMM-FFI group, the maximum value of HDL and the minimum value of the triglyceride level were revealed against the background of the use of the developed FFIs. In the IMM-FFI group, there was a significant decrease in ALT and an increase in AST levels compared with the animals of the IMM group, against the background of no differences in the IMM-FFI group compared with the CTRL group. Thus, the consumption of FFIs contributes to the compensation of transaminase levels in rats. The detected increase in blood glucose levels in animals of the IMM-FFI group is within the range of fluctuations in the physiological norm for rats of this line and this age. Nevertheless, the presence of such significant changes indicates a decrease in the intensification of glycolysis processes.

The biochemical parameters of rat urine (Table 4) revealed a significant increase in phosphorus excretion after exposure to daily immobilization in the experimental groups IMM and IMM-FFI. There were no changes between the groups in the urinary excretion of prostaglandin E2.

**Figure 4:**
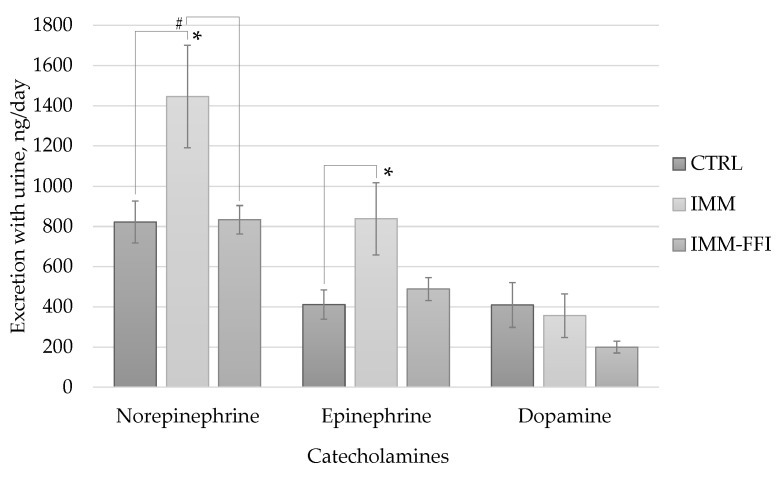
Daily urinary catecholamine excretion, ng/day. *—differences are significant compared with the CTRL group, *p* < 0.05. #—differences are significant compared with the RUN group, *p* < 0.05.

Figure 4 shows the results of determining the daily excretion of catecholamines in urine. The daily forced immobilization of animals of the IMM group led to a significant increase in the daily urinary excretion of catecholamines—norepinephrine and epinephrine. However, there were no significant changes in urinary catecholamine excretion levels in the IMM-FFI group. Thus, the consumption of FFI by animals contributed to the normalization of epinephrine and norepinephrine levels despite daily immobilization stress.

The results of the evaluation of biochemical parameters characterizing the antioxidant status of the rat organism are presented in Table 5. In the IMM group, where rats were subjected to immobilization stress, the amount of malondialdehyde increased by more than 77% compared with the CTRL group. The introduction of FFIs into the diet of animals reduced this indicator to the level of the CTRL group. In addition, in animals of the IMM-FFI group, 24 h after stress, there was a significant increase in SOD activity by 35% compared with animals of the CTRL group.

## 3. Discussion

The plant polyphenols of *Chenopodium quinoa* represent an extensive heterogeneous group of compounds with a variety of physiological and biochemical effects in living systems. Quinoa saponins are characterized by very uncertain properties, manifested by both antimicrobial and anti-inflammatory activities on the one hand and hemolytic and cytotoxic activities on the other [23,24]. Despite the limited acute toxic effect of crude saponins of quinoa leaf husk [25], they are considered an antinutritional factor, which requires their removal before consumption [23]. Some other polyphenolic compounds included in quinoa may also exhibit cytotoxic properties and have carcinogenic or genotoxic effects in high doses or concentrations [26]. The technological scheme we have chosen for the production of FFIs allows us to exclude saponins from the composition and concentrate phytoecdysteroids 20E in *Chenopodium quinoa* more than 200 times in the final product compared with the raw material [27]. A single oral administration to mice of the FFIs developed by us from *Chenopodium quinoa* at a dose of 5000 mg/kg of body weight did not lead to the death of animals, did not contribute to the development of pathological changes in internal organs, and did not affect body weight gain, relative liver weight, lipid profiles, or levels of biochemical parameters in the blood after 14 days of observation. The 36-day experiment, despite the presence of daily episodes of stress caused by forced immobilization, also demonstrated a positive safety profile of FFIs in the studied dose for the determined indicators. The results obtained allow us to classify the FFIs from *Chenopodium quinoa* as a low-hazard substance [22].

The use of laboratory rodents in stress modeling [28,29] allows us to reproduce the scenario of emotional stress [30], the advantage of using which, compared with humans, is the behavioral instinctiveness of rodents, depending on the model. In preclinical studies of the adaptogenic properties of BASs, a necessary condition is the exclusion of factors of the heterogeneity of stress states in humans. The model of immobilization stress in rodents causes the activation of the hypothalamic-pituitary-adrenal axis and increased levels of glucocorticoid hormones, leading to the dysfunction of the glucose metabolism, oxidative-nitrosative stress, and oxidative tissue damage [31,32]. Restraint stress in rats leads to a decrease in memory function, the development of anxiety-like functions, and an increase in motor activity, changing the functional state of antioxidant enzymes in the brain, especially in the poststress period [33,34].

We have previously shown that immobilization has a strong stressful impact on male Wistar rats, leads to the increased excretion of catecholamines and circulating corticosterone levels, reduced energy consumption, and, as a consequence, a lag in body weight gain [33]. One of the components influencing weight loss during immobilization stress, apparently, is the increased expression kinetics of anorexigenic (POMC and CART) and orexigenic (NPY and AgRP) peptides [35] and, as a result, anorexia caused by a violation of the homeostatic circuit [36]. In the present study, the average cumulative feed intake in stressed animals of the IMM and IMM-FFI groups did not differ and was also lower compared with the CTRL group; however, the introduction of FFIs into the diet had a positive effect on the body weight of animals up to the 25th day of the experiment, despite the hypolipidemic properties of quinoa [12,13,14]. The consumption of FFIs neutralized to some extent the negative impact of immobilization on the energy metabolism of animals subjected to stress.

The peculiarities of animal behavior in the combined use of two physiological tests, EPM and OF, showed that the inclusion of FFIs in the diet showed pronounced anxiolytic and antidepressant properties, thus leveling the effect of immobilization stress. This was also manifested in the improvement of long-term memory indicators in animals in the CRPA test and the normalization of the levels of central neurotransmitters—epinephrine and norepinephrine, mediating stress effects through the pituitary-adrenal system during the formation of free radicals [37]. Under immobilization stress, there is a significant increase in acetylcholinesterase activity in some parts of the rat brain [34]. The central cholinergic system also plays an important role in the regulation of learning and memory, which are key components of cognitive behavior. In [38], the ability of the water-alcohol extract of quinoa grain to inhibit the activity of acetylcholinesterase was shown. A decrease in acetylcholinesterase activity under stress can improve cognitive functions by inducing cholinergic activity by increasing acetylcholine levels [37]. The ability to maintain pro-/antioxidant homeostasis in the cerebral cortex and hippocampus in mice under immobilization stress has been demonstrated by protocatechuic acid, which is part of quinoa composition [39]. A pronounced anxiolytic effect mediated by the GABAergic neurotransmitter system was detected in sinapinic acid, also present in the polyphenolic profile of quinoa [8,15].

The nutritional support of FFIs contributed to hypolipidemic action in rats, some compensation of the circulating transaminases activity, and normalization to control values of malondialdehyde levels, the concentration of which increases in all brain structures during immobilization stress, reflecting the rate of the formation of reactive oxygen species [34]. Another key component of the antioxidant system is the enzyme superoxide dismutase (SOD), which participates in the elimination of excess superoxide. The suppression of SOD activity indicates an increase in the production of superoxide, an overabundance of which negatively affects the functional abilities of neurons in the cerebral cortex. The increase in the activity of SOD revealed in our study 24 h after stress exposure against the background of animal consumption of FFIs suggests that its main components, namely 20-hydroxyecdysone and polyphenols, can act as suppressors and activate the antioxidant defense system. The component 20-hydroxyecdysone is able to mediate its neuroprotective effect by activating cellular antioxidant enzymes, inhibiting oxidative stress, and reducing the levels of reactive oxygen species [40].

The use of scenarios that feature the preventive use of FFIs on alternative in vivo stress models involving molecular-genetic and proteomic approaches to assess the functional state of nuclear transcription factors, c-Jun N-terminal kinases, and signaling of the intracellular antioxidant defense system of neurons will shed light on the understanding of the mechanisms of relieving anxiety and depression by polyphenolic compounds under stress.

## 4. Materials and Methods

### 4.1. Preparation and Characterization of the FFIs from Chenopodium quinoa Seeds

**Figure 5:**
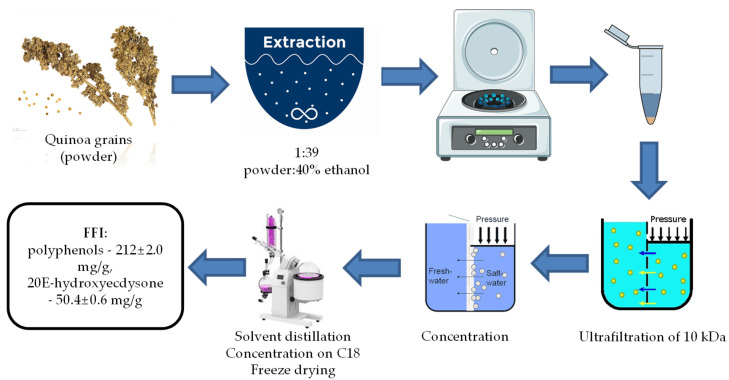
Flow chart of the FFI production from *Chenopodium quinoa*.

We used commercial black quinoa (*Chenopodium quinoa*) grains preliminarily ground in a laboratory blender (FimarFRI, Rimini, Italy) and sieved to obtain a particle size of less than 0.35 mm. The detailed extraction procedure, FFI production method, and FFI polyphenolic composition are presented in our previously published work [27]. Phytoecdysteroid (20E) content was determined by HPLC-MS. The total polyphenol content was determined by the Folin-Ciocalteu method. Total 20E content in the composition of FFIs was 50.4 ± 0.6 mg/g. Total flavonoid content was 212.0 ± 2.0 mg/g. The technological scheme of FFI production is presented in Figure 5.

### 4.2. Animals and Experimental Design

#### 4.2.1. Animals and Ethics

Male Wistar rats were used in the experiments on the evaluation of FFI effectiveness against stress. ICR (CD) mice were used in the experiments on the evaluation of the acute oral toxicity of FFIs. Animals were purchased in the Stolbovaya nursery (a scientific center of biomedicine technologies of a federal medical-biological agency, Moscow region, Russia). Animals were housed in polycarbonate cages, two per cage under the following environmental conditions: temperature 21–24 °C, relative humidity 30–60%, 12/12 h day/night cycle. Animals were fed with a balanced half-synthetic diet according to AIN93M [41] and water, filtered in reverse-osmosis apparatus (Merck-Millipore, Berlington, MA, USA). All animal procedures were conducted according to standard requirements [42]. Animal studies were approved by the Ethics Committee of the Federal Research Centre of Nutrition and Biotechnology (protocol code No. 02-19, 6 October 2019) in accordance with the standard principles described in “Guide for the Care and Use of Laboratory Animals”.

#### 4.2.2. Acute Oral Toxicity: Fixed Dose Procedure

The experiment on the evaluation of the acute oral toxicity of FFIs was conducted according to OECD Guidelines for the Testing of Chemicals No. 425 [22], with some modifications. The experiment used 16 male and 16 female mice (healthy adult animals, age—5 weeks) with an initial body weight 26 ± 2 g and 24 ± 1 g, respectively. After 1 week of acclimatization, animals were divided into 4 groups (n = 8): 1—control males, 2—experimental males, 3—control females, and 4—experimental females. Experimental animals were fed by gavage with FFIs in a dose of 5000 mg/kg of body weight. FFIs were dissolved in drinkable water and filtered by reverse osmosis. Control animals were gavaged by water in the same volume. Animals were monitored for 6 h after FFI administration. General condition, motor activity, convulsion, skeletal muscle tonus, breath rate, breath depth, fur quality, mucous coat condition, feces, and urination were monitored. In the 13 days following, all these parameters were still monitored. On the 14th day, animals were euthanized in a CO_2_ chamber. The autopsy was conducted, and the condition of thoracic and abdominal cavities organs was studied. The relative liver weight was determined; cholesterol and triglyceride levels were determined in the liver; and corticosterone and prostaglandin E2 levels were determined in the blood.

#### 4.2.3. 36-Day Study of the Quinoa FFI Adaptogenic Properties in a Model of Immobilization-Induced Emotional Stress

Sixty male Wistar rats with an initial body weight 130 ± 2 g were used for this experiment (healthy animals, age—5 weeks). Adaptation reactions to stress differ between animals according to their behavior. The behavioral phenotype of animals was studied before the experiment to divide them into active and passive species. The preliminary division of animals according to their behavior increases the repeatability of obtained results [43,44]. To achieve that, after 7-day quarantine, rats were divided according to their behavior in the elevated plus maze test (EPM). Animals were tested for 5 min. The number of zone transitions, time in open and closed arms of the maze, and total motor activity were registered. According to test results, 36 of the 60 animals were chosen for the main experiment. To evaluate the changes in the anxiety-like functions level of animals, the test was repeated on the 28th day of the experiment. The rat activity in the maze was monitored with the use of a Smart 3.0.04 video-tracking system (Panlab Harvard Apparatus, Barcelona, Spain). The detailed characteristics of the used EPM and anxiety-like functions evaluation method were described earlier, in [45].

Animals were randomly divided (according to the body weight and EPM test results) into three groups (n = 12): CTRL, IMM, and IMM-FFI. Animals of the CTRL group and IMM group were treated during 36 days of experiment with a standard half-synthetic diet. The rats of the third experimental group, IMM-FFI, were treated with a standard diet with the addition of FFIs in the dose of 0.055 ± 0.003%. All diets were isocaloric and isonitrogenous. Animals received food and water ad libitum; food consumption was monitored three times a week. Body weight gain was monitored twice a week. Animals in the IMM and IMM-FFI groups during the whole experiment were exposed to forced immobilization. Rats were put into animal restrainers (AE1001-R1, Open Science, Russia), limiting their freedom of movement. The duration of daily immobilization was 40 min.

In the study, training, short-term memory, and long-term memory were evaluated using conditioned passive avoidance test (CPAR), using methods and apparatus described earlier, in [46]. The test was conducted on the 14th day of the experiment (training), on the 15th day (short-term memory), and on the 29th day (long-term memory).

The ability to research behavior reflecting anxiety-like functions and the desire of rats to explore a new territory was evaluated on the 22nd day of the experiment in the open field test using the methodology and equipment described earlier, in [46]. The animals were tested during the periods of their minimal daily activity from 10-00 am to 3-00 pm.

On the 36th day of the experiment, the animals in the IMM and IMM-FFI groups were exposed to exhausting immobilization for 180 min. Immediately after the immobilization, the animals were moved into metabolic cages for urine collection.

**Figure 6:**
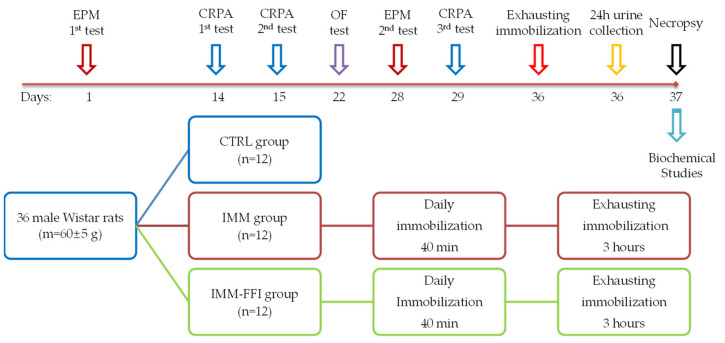
Design and timeline of the experiment.

In 24 h on the 37th day, animals that fasted overnight were decapitated under light ether anesthesia. Blood was collected, centrifugated for 15 min at 4000 rpm, and kept at −80 °C for further analysis. Catecholamine content was determined in urine, and parameters of protein, lipid, and mineral metabolism, functional liver condition, and the antioxidant system were determined in the blood. The experimental design is presented in the Figure 6.

### 4.3. Methods for Assessing Biochemical Parameters

The parameters of protein metabolism (total protein, urea, creatinine), lipid metabolism (total cholesterol, LDL, HDL, triglycerides), purine metabolism (uric acid), mineral metabolism (phosphorus, magnesium, calcium), functional liver condition (total bilirubin, ALT, AST, alkaline phosphatase), and glucose level were determined in blood plasma; total urea, uric acid, creatinine, magnesium, phosphorus, and calcium content in urine were determined using Konelab 20i automatic biochemical analyzer (Thermo Scientific, Waltham, MA, USA). Superoxide dismutase (Cayman Chemical, Ann Arbor, MI, USA), malondialdehyde and glutathione reductase (Cloud-Clone Corporation, Houston, TX, USA), catalase (Cayman Chemical, Ann Arbor, MI, USA), and corticosterone (IDS Limited, Bramham, UK) content were determined in blood plasma using commercial ELISA kits.

Prostaglandin E2 urinary content was determined by the ELISA method using a commercial kit according to manufacturer instructions (R&D systems, Minneapolis, MN, USA). Norepinephrine, epinephrine, and dopamine urinary contents were determined using liquid chromatograph Agilent 1100 (Agilent Technologies, Santa Clara, CA, USA) with a Thermo TSQ triple-quadrupole mass spectrometer detector with electrospray ionization, an autosampler, and software for processing chromatographic data, specifically Chem Station and Endura. HPLC conditions were as follows: Thermo Acclaim PolarAdvantage II column (3 µm, 150 × 3 mm); gradient elution in the Milli-Q water system with the addition of 0.1% formic acid (A)—acetonitrile with the addition of 0.1% formic acid (B) (0–6 min, 2–20% B; 6–7 min, 20–20 V, 7–15 min–2% B); column temperature—30 °C; eluent flow rate, 0.5 mL/min; and the volume of the injected sample is 5 µL. Mass scanning was performed in the positive ion detection mode in the range of *m*/*z* 50–500. Operating parameters of the ionization source were as follows: capillary voltage—3.5 kV, drying gas flow (nitrogen)—10 L/min, temperature—325 °C, and pressure on the sprayer—50 psig. Solutions in methanol of individual substances were used for the analysis: dopamine hydrochloride, norepinephrine tartrate, and adrenaline. Retention times were as follows: norepinephrine (1.81 min), epinephrine (1.99 min), and dopamine (2.15 min). As standards for catecholamines and an internal standard (3,4-dihydrobenzylamine hydrobromide), preparations manufactured by Sigma-Aldrich (US) with a substance content of 98–99% were used. Sample preparation of the test samples was carried out as follows: urine (1 mL) was centrifuged (4000 rpm, 30 min), then 1.0 M Tris-HCl buffer (pH 8.6) was adjusted to pH 8.5–8.7 according to the universal indicator, and 40 µL of an internal standard solution, 3,4-dihydrobenzylamine hydrobromide (SIGMA—ALDRICH, Saint Louis, MI, USA), was quantitatively applied to a microcolumn (0.5 × 1.0 cm) with alumina (neutral according to Brockmann). The sorbent was washed with distilled water (2 × 2 mL), and catecholamines were eluted with 400 µL of 1.0 M acetic acid solution.

### 4.4. Statistical Analysis

The statistical analysis of obtained data was processed using the SPSS Statistics 24 program (IBM, US) and Microsoft Excel for Windows. Mean (M), standard deviation (SD), and standard error of the mean (m) were calculated. The probability of accepting the null hypothesis about the coincidence of the distributions of the compared samples was determined using the two-tailed Student’s t-test for pairwise related values, the Wilcoxon-Mann-Whitney nonparametric post hoc test, the Kruskal-Wallis test, and ANOVA. Data are presented as M ± s.e.m. The critical significance level of the null hypothesis (*p*) was taken as equal to 0.05.

## 5. Conclusions

The conducted experiment demonstrated the effectiveness of nutritional support using FFIs from *Chenopodium quinoa* on the immobilization stress model. The use of FFIs under conditions of induced oxidative/nitrosative stress normalized the levels of malondialdehyde and catecholamines against the background of maintaining the high activity of superoxide dismutase, thus contributing to the normalization of the behavioral reactions of animals and their cognitive functions. Because of the absence of gluten proteins, gliadins and protein fractions corresponding to gliadin, it is advisable to include FFIs from quinoa in a personalized diet as a component of specialized foods for patients with celiac disease. The absence of such antinutritional factors as saponins and the positive safety profile of FFIs makes it advisable to further study the adaptogenic properties of FFIs in clinical studies in the composition of specialized foods in various categories of people exposed to chronic stress.

## Figures and Tables

**Table 1:** Group formation according to the EPM test and body weight of rats.

Indicator	Group		
	CTRL	IMM	IMM-FFI
Body weight, g	130 ± 3	129 ± 4	131 ± 3
Time in open arms, s	49 ± 7	51 ± 10	40 ± 9
Time in closed arms, s	225 ± 9	214 ± 15	237 ± 12
Distance covered, cm	1103 ± 72	1248 ± 144	1175 ± 129
Number of transitions	19 ± 2	23 ± 4	21 ± 3

**Table 2:** Behavioral reactions of rats in the CRPA test.

Group	First Test CRPA Formation		Second Test after 24 h Short-Term Memory		Third Test after 14 Days Long-Term Memory	
	Latency, s	Entered Animals, %	Latency, s	Number of Animals Entered	Latency, s	Number of Animals Entered
CTRL	18 ± 3	100	117 ± 22	5 (41%)	43 ± 15	11 (92%)
IMM	27 ± 8	100	156 ± 16	2 (16%)	75 ± 22	8 (67%)
IMM-FFI	23 ± 4	100	126 ± 23	4 (33%)	88 ± 23	7 (58%)

**Table 3:** Biochemical blood indicators of rats.

Indicator	Group		
	CTRL	IMM	IMM-FFI
HDL, mmol/L	0.83 ± 0.05	0.96 ± 0.05	1.00 ± 0.04 ^1^
LDL mmol/L	0.23 ± 0.02	0.23 ± 0.01	0.20 ± 0.01
Triglycerides, mmol/L	1.09 ± 0.12	0.90 ± 0.05	0.82 ± 0.03 ^1^
Cholesterol, mmol/L	2.03 ± 0.10	2.17 ± 0.08	2.20 ± 0.11
AlAT, U/L	77.8 ± 4.5	89.1 ± 3.4	75.6 ± 2.6 ^2^
AsAT, U/L	185.1 ± 17.5	131.2 ± 23.9	211.0 ± 14.3 ^2^
Total bilirubin, mmol/L	4.73 ± 0.31	6.07 ± 0.50 ^1^	5.57 ± 0.52
Total protein, g/L	65.7 ± 0.9	65.0 ± 0.5	63.7 ± 0.6
Phosphorus, mmol/L	2.90 ± 0.05	2.96 ± 0.06	2.92 ± 0.05
Calcium, mmol/L	2.64 ± 0.02	2.61 ± 0.02	2.63 ± 0.02
Magnesium, mmol/L	0.96 ± 0.03	1.01 ± 0.04	0.96 ± 0.03
Alkaline phosphatase, U/L	342 ± 25.3	349 ± 20.6	391 ± 35.1
Urea, mmol/L	5.25 ± 0.11	5.06 ± 0.07	5.05 ± 0.12
Uric acid, mmol/L	86.29 ± 6.1	98.27 ± 4.4	87.09 ± 4.3
Creatinine, mmol/L	25.04 ± 0.65	26.41 ± 1.00	25.37 ± 0.68
Glucose, mmol/L	5.29 ± 0.45	5.06 ± 0.20	5.93 ± 0.16 ^2^
Corticosterone, ng/mL	19.2 ± 4.4	48.2 ± 10.5 ^1^	56.4 ± 9.4 ^1^

Note: ^1^—differences are significant against CTRL group; ^2^—differences are significant against IMM group, *p* < 0.05. HDL—high-density lipoprotein; LDL—low-density lipoprotein; AlAT—alanine aminotransferase; AsAT—aspartate aminotransferase.

**Table 4:** Biochemical parameters of urine.

Indicator	Group		
	CTRL	IMM	IMM-FFI
Calcium, µg/day	5.5 ± 1.2	5.8 ± 1.8	4.9 ± 0.7
Creatinine, mg/day	44.2 ± 3.3	43.5 ± 2.1	44.7 ± 1.7
Magnesium, µg/day	27.0 ± 2.8	35.2 ± 2.7	33.7 ± 3.1
Uric acid, mg/day	21.9 ± 1.7	23.8 ± 1.4	22.2 ± 0.9
Urea, mg/day	3.9 ± 0.7	5.3 ± 0.5	5.1 ± 0.3
Phosphorus, µg/day	560.5 ± 38.7	695.5 ± 40.1 ^1^	709.4 ± 46.2 ^1^
Prostaglandin E2, ng/day	20.1 ± 2.0	19.3 ± 3.3	19.9 ± 2.5

Note: ^1^—differences are significant against CTRL group, *p* < 0.05.

**Table 5:** Indicators of the antioxidant status of rats’ blood.

Indicator	Group		
	CTRL	IMM	IMM-FFI
Malondialdehyde, mg/mL	9.7 ± 1.5	17.2 ± 3.6 ^1^	11.4 ± 1.6
Glutathione reductase, ng/mL	113.2 ± 10.5	93.6 ± 7.0	96.9 ± 7.3
Superoxide dismutase, units/mL	1755.7 ± 92.9	1997.1 ± 236.5	2369.6 ± 225.4 ^1^

Note: ^1^—differences are significant against CTRL group, *p* < 0.05.

## Data Availability

Data are available on request because of restrictions: privacy or ethical.

## Supplementary Materials

The following are available online at https://www.mdpi.com/article/10.3390/molecules27249003/s1. Table S1: Determination of acute toxicity of quinoa FFI. Health monitoring indicators up to 240 min after administration. Table S2: Biochemical parameters of the blood and liver of animals in the experiment of quinoa FFI acute toxicity assessment.
